# Efficient, high-performance semantic segmentation using multi-scale feature extraction

**DOI:** 10.1371/journal.pone.0255397

**Published:** 2021-08-19

**Authors:** Moritz Knolle, Georgios Kaissis, Friederike Jungmann, Sebastian Ziegelmayer, Daniel Sasse, Marcus Makowski, Daniel Rueckert, Rickmer Braren

**Affiliations:** 1 Department of Diagnostic and Interventional Radiology, Klinikum rechts der Isar, School of Medicine, Technical University of Munich, Munich, Germany; 2 Institute for Artificial Intelligence and Informatics in Medicine, Klinikum rechts der Isar, School of Medicine, Technical University of Munich, Munich, Germany; 3 OpenMined; 4 Department of Computing, Imperial College London, London, United Kingdom; Universita degli Studi di Pisa, ITALY

## Abstract

The success of deep learning in recent years has arguably been driven by the availability of large datasets for training powerful predictive algorithms. In medical applications however, the sensitive nature of the data limits the collection and exchange of large-scale datasets. Privacy-preserving and collaborative learning systems can enable the successful application of machine learning in medicine. However, collaborative protocols such as federated learning require the frequent transfer of parameter updates over a network. To enable the deployment of such protocols to a wide range of systems with varying computational performance, efficient deep learning architectures for resource-constrained environments are required. Here we present *MoNet*, a small, highly optimized neural-network-based segmentation algorithm leveraging efficient multi-scale image features. *MoNet* is a shallow, *U-Net*-like architecture based on repeated, dilated convolutions with decreasing dilation rates. We apply and test our architecture on the challenging clinical tasks of pancreatic segmentation in computed tomography (CT) images as well as brain tumor segmentation in magnetic resonance imaging (MRI) data. We assess our model’s segmentation performance and demonstrate that it provides performance on par with compared architectures while providing superior out-of-sample generalization performance, outperforming larger architectures on an independent validation set, while utilizing significantly fewer parameters. We furthermore confirm the suitability of our architecture for federated learning applications by demonstrating a substantial reduction in serialized model storage requirement as a surrogate for network data transfer. Finally, we evaluate *MoNet*’s inference latency on the central processing unit (CPU) to determine its utility in environments without access to graphics processing units. Our implementation is publicly available as free and open-source software.

## Introduction

Access to large collections of data remains one of the key challenges in successfully applying machine learning to many problems in medicine. Common machine learning datasets, such as ImageNet [1] with >1 million images, are much larger than their counterparts used in medical studies. Even large recent studies [2, 3] use datasets significantly smaller than ImageNet and orders of magnitude smaller than the datasets used to train state-of-the-art language models [4]. Furthermore, current medical studies often source data from only few institutions, thus preventing the training of representative and unbiased models, suitable for application in a broad variety of patient collectives [5]. Algorithms trained on single-institutional data have recently been shown to cause generalization challenges to out-of-sample data [6]. One of the main hindrances to large-scale, multi-institutional medical data collection, which could address this challenge, is the strict regulation of patient data, preventing its exchange and mandating the development of decentralized learning systems [7].

Federated machine learning [8] allows for collaborative training of algorithms on data from different hospitals (*data silos*) or edge devices (such as wearable health sensors or mobile phones) without the need for central aggregation of said data. In federated learning, a model is trained in a distributed fashion. Individual models are trained locally on data which never leaves a participating site (*node*), and only parameter updates are sent via the network to be aggregated by the coordinating node (*hub-and-spoke topology*). Federated learning enhanced by privacy-preserving techniques [9] such as differential privacy [10] holds the promise of secure, large-scale machine learning on confidential, medical data.

The utilization of federated learning techniques on the largest possible number of institutions and patients from a diverse geographic, demographic and socio-economic background will require the development of systems suitable for execution on a broad range of hardware including mobile devices and systems without graphics processing units, which may be too expensive for deployment e.g. in the developing world. A further key component of this democratization is the improvement of system efficiency, as federated learning requires the frequent transfer of parameter updates over a network. Previous work [11] has mainly focused on improving communication efficiency in federated learning by compression of parameter updates or sophisticated update aggregation methods [12, 13]. Other works have focused on increasing the computational efficiency of model architectures: The MobileNet family of models [14] utilizes depth-wise separable convolutions and a reduced parameter count to achieve this goal and target deployment on edge computing/mobile devices. The EfficientNet model architectures [15] recently proposed attempts to achieve optimal trade-offs between input resolution, network depth and width for classification performance. However, the targeted design of small and efficient neural network architectures for the specific task of semantic segmentation has so far remained under-explored. To the contrary, deep learning-based segmentation has focused on expanding model size with large ensembles of neural networks [16], rendering them impractical for deployment in the federated setting.

Here, we introduce *MoNet*, a very small, shallow, *U-Net*-derived semantic segmentation architecture based on efficient multi-scale feature extraction using repeated decreasingly dilated convolution (RDDC) layers with two global down-sampling operations and a total of 403,556 parameters. We showcase our architecture’s performance on the challenging task of pancreatic segmentation as well as brain tumor segmentation and demonstrate substantial efficiency gains and segmentation performance competitive with much larger models.

## Methods

### Training, validation and independent testing datasets

**Fig 1:**
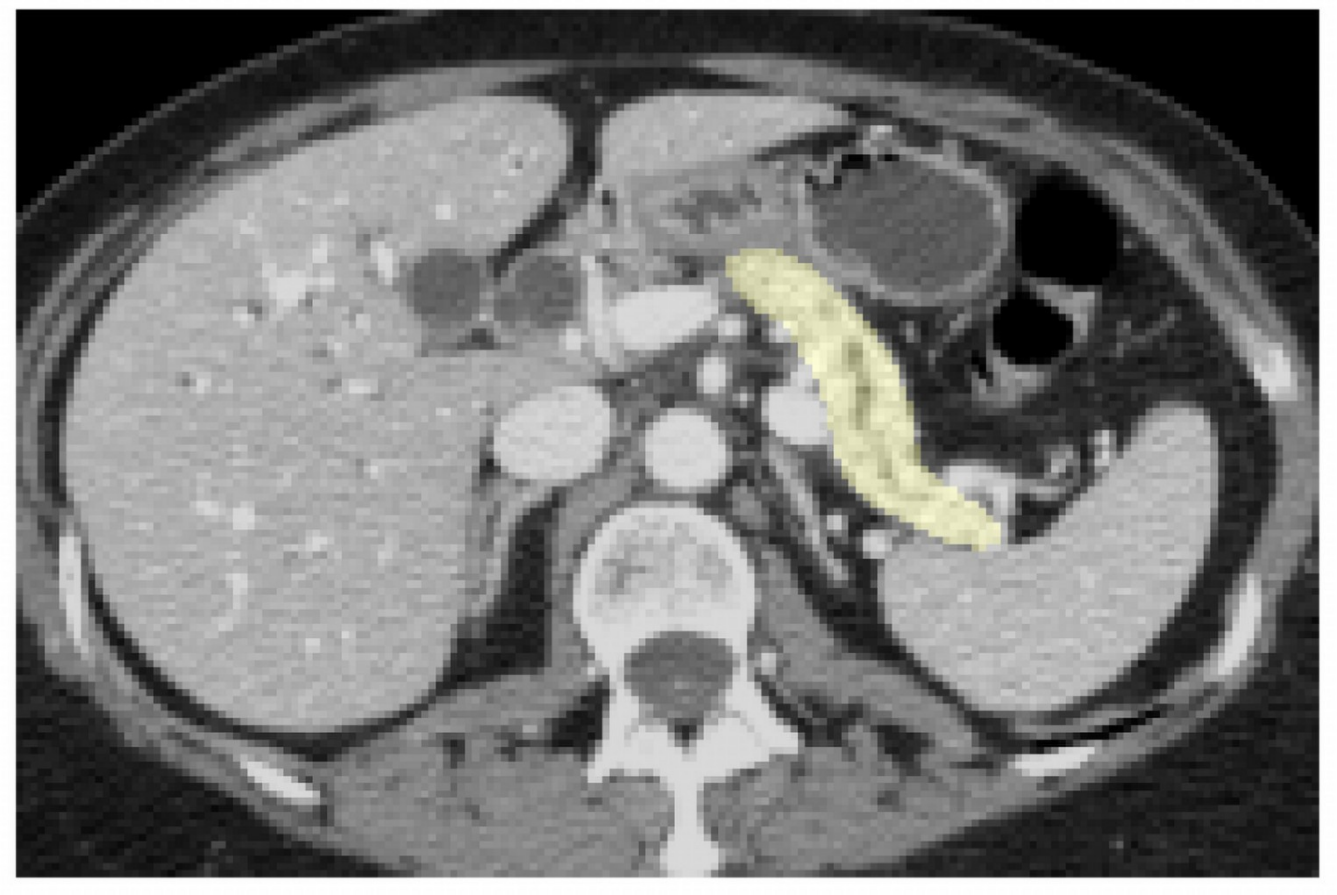
Axial slice of a ground truth pancreas segmentation in an abdominal CT scan (MSD), cropped to show detail of surrounding tissues.

All neural network architectures presented in this work were trained two different datasets from the Medical Segmentation Decathlon (MSD) [17]: pancreas and brain tumor segmentation. A random, consistent 70%/30% training-validation split was employed for both datasets. For processing, images were bilinearly down-sampled to 256 × 256, and the segmentation labels were merged yielding a binary segmentation task. To assess out-of-sample generalization performance on the pancreas dataset, independent validation of the architectures was performed on an unseen, clinical PDAC dataset consisting of 85 abdominal CT scans in the portal-venous phase collected at our institution. For the brain tumor dataset, no in-house clinical dataset was available. All clinical data were collected according to Good Clinical Practice and in consent with the Declaration of Helsinki. The use of imaging data was approved by the institutional ethics committee (Ethikkommission der Fakultät für Medizin der Technischen Universität München, protocol number 180/17S, May 9th 2017) and the requirement for informed written consent was waived. The pancreas including the tumor was manually segmented by a third-year radiology resident, then checked and corrected as necessary by a sub-specialized abdominal radiologist. An exemplary ground truth label mask superimposed on a CT slice from the training set is shown in Fig 1.

### Network architecture

**Fig 2:**
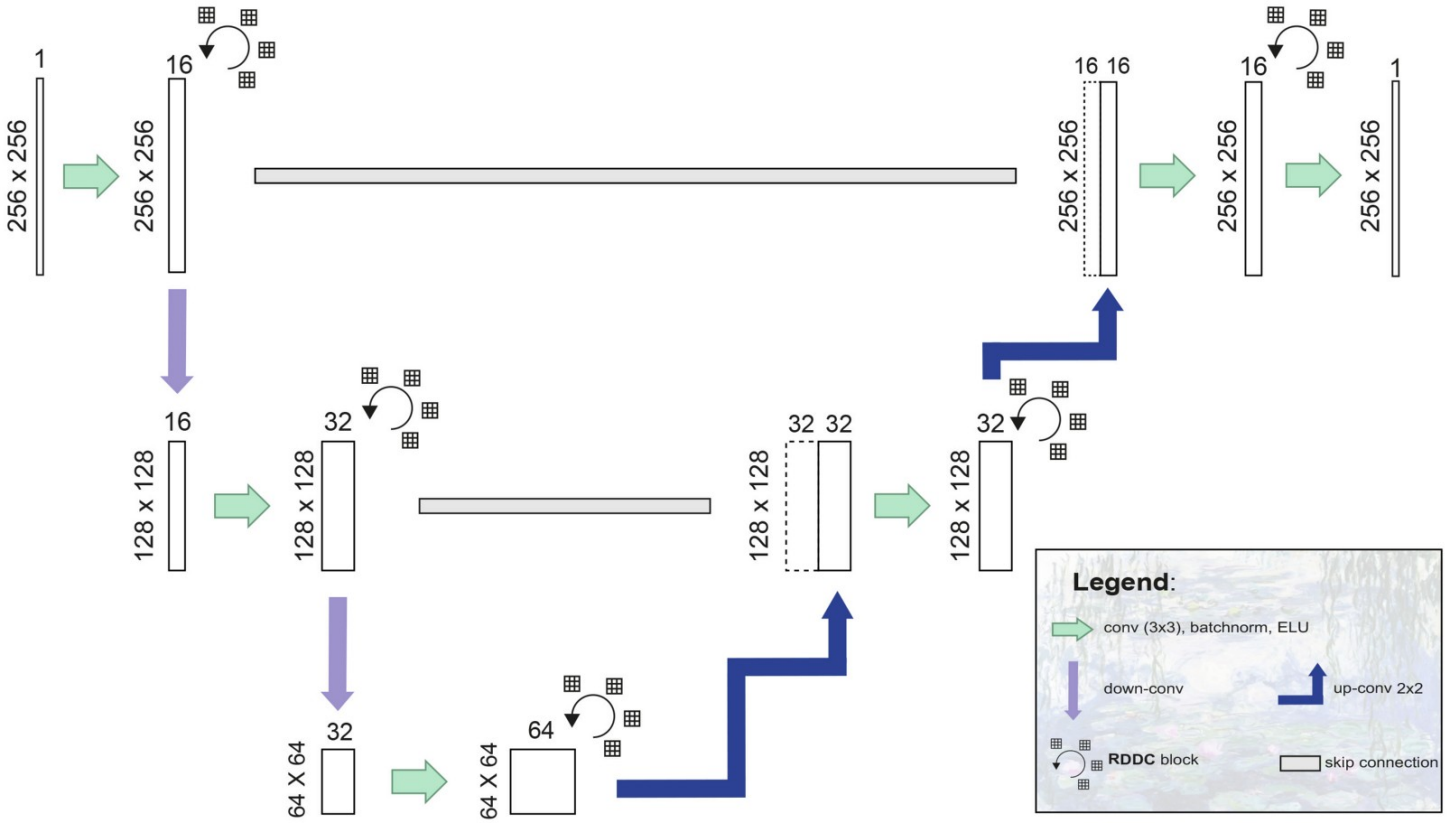
Schematic representation of the *MoNet* architecture.

**Fig 3:**
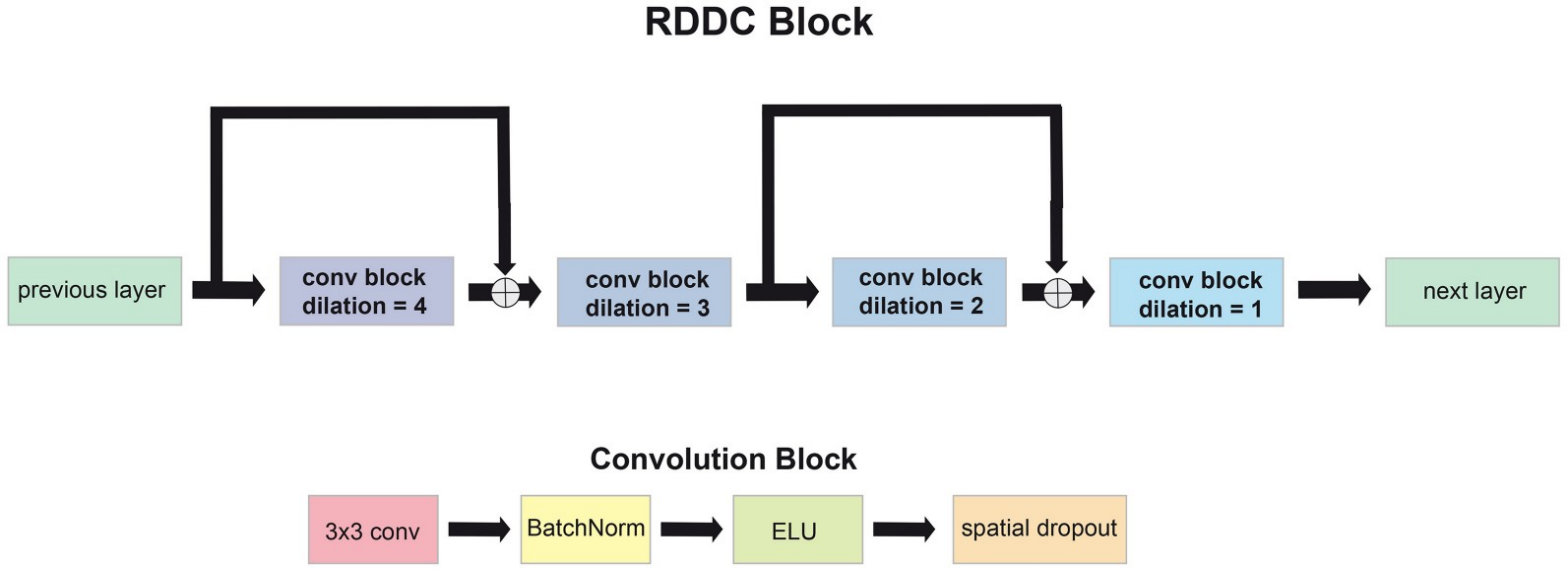
Schematic representation diagram of a *RDDC* block (top) and the constituent convolutional (bottom).

The architecture of *MoNet* is depicted in Fig 2. In brief, 4-dimensional input tensors of shape *B* × 256 × 256 × 1, with *B* denoting the batch size, are progressively down-sampled across the encoder branch of the network using convolutions with a stride length of 2, resulting in an *X* × *Y* resolution of 64 × 64 in the *bottleneck* segment of the network. The resulting feature maps are then progressively up-sampled by transposed convolution (*de-convolution*) in the decoder branch resulting in output masks of identical dimensions as the input. Each (de-)convolution block consists of a 3x3 convolutional layer followed by batch normalization and an *exponential linear unit* (ELU) activation. At every stage in the *U-Net*-like architecture, the convolution blocks are followed by a *repeated decreasingly dilated convolution* (RDDC) block (Fig 3), consisting of four successive convolutional blocks as described above, but employing dilated convolutions [18] with a decreasing dilation rate (4, 3, 2, 1, respectively). This feature extraction strategy has been shown to perform well for small objects [19]. Each convolutional block within a *RDDC* block is followed by a spatial dropout layer [20]. Finally, residual-type longitudinal (short) connections are employed within each RDDC block and transverse (long) skip connections are employed between the encoder and the decoder branch to assist signal and gradient flow as originally described in [21, 22].

### Model training

All architectures were trained to convergence using the *Nesterov-Adam* optimizer [23] with an initial learning rate of 5 × 10^−4^ and learning rate decay by a factor 10 upon validation loss stagnation for ≥ 2 epochs. Weights were initialized using uniform He-initialization [22] and the Dice loss [24] was used to train all networks. Data augmentation was used in the form of random rotations up to 10 degrees, random zoom (±0.25) and random pixel shifts of a maximum magnitude of 0.2 of the image height/width. All architectures were trained to segment the entire pancreas including the tumor. This approach is owing to the fact that the exact delineation of the tumor border is often times infeasible and supported by literature findings noting the importance of the peritumoral tissue in PDAC [25–27] and in other tumor entities [28]. To maintain comparability, we also merged the labels in the brain tumor dataset to obtain a binary segmentation task.

### Performance assessment

We compared *MoNet*’s performance to the following three *U-Net* baselines:

- original *U-Net* [21], 64 base filters (*U-Net-64*)
- original *U-Net* [21], 16 base filters (*U-Net-16*)
- Attention-gated *U-Net* [29], 2D, 64 base filters (*Attention U-Net*)

## Results

### Segmentation performance comparison

**Fig 4:**
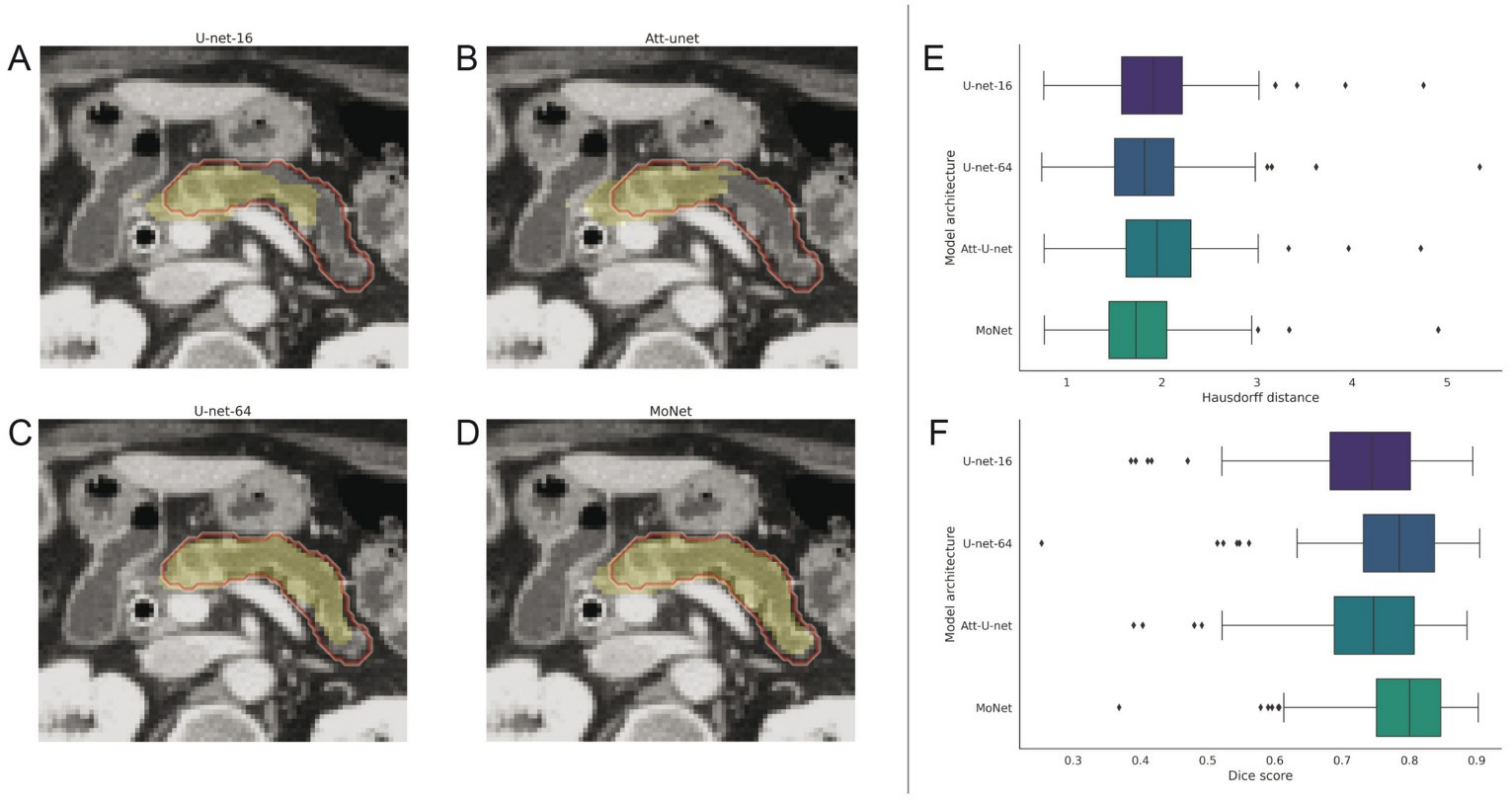
Exemplary segmentation results (yellow) of: *U-Net-16* (A), *Attention U-Net* (B), *U-Net-64* (C), *MoNet* (D), on the pancreas MSD validation set, Ground truth indicated by red outline. Box-plots of Hausdorff distances (E) and Dice scores (F) computed for the whole pancreas MSD validation set on a per-patient basis.

*MoNet* performed similarly or on par with other *U-Net* variants on the validation dataset (pancreas & brain tumor) while outperforming the other *U-Net* variants in out-of-sample generalization on the independent validation dataset (pancreas only). Results are summarized in Table 1 and visualized in Fig 4.

**Table 1:** Comparison of *MoNet* with other *U-Net* variants in two different imaging modalities on the task of pancreas and brain lesion segmentation, CT and MRI respectively. We report performance on validation sets of the MSD datasets (brain tumor and pancreas) as well as out-of sample generalization performance on an independent validation set (IVD), collected and annotated in-house.

Task	Architecture	Dice Score, MSD	Hausdorff Distance, MSD	Dice Score, IVD
Pancreas	*U-Net-64*	0.76±0.11	1.86±0.66	0.50±0.2
Pancreas	*U-Net-16*	0.73±0.10	1.96±0.66	0.59±0.2
Pancreas	*Attention U-Net*	0.73±0.10	1.98±0.66	0.37±0.6
Pancreas	*MoNet (ours)*	**0.78±0.090**	**1.78±0.61**	**0.70±0.1**
Brain Tumor	*U-Net-64*	**0.78±0.12**	**2.01±0.38**	—
Brain Tumor	*U-Net-16*	0.77±0.12	2.03±0.38	—
Brain Tumor	*Attention U-Net*	0.74±0.15	2.09±0.38	—
Brain Tumor	*MoNet(ours)*	0.77±0.13	2.04±0.39	—

### Training speed & inference time comparison

To compare the performance of *MoNet* in a typical inference setting on CPU, as well as when performing GPU training. We recorded the time required for doing inference with 150 256 × 256 images on CPU (2.4GHz 8-Core Intel Core i9) and the time per batch when training on GPU (batch size = 32). All experiments were performed with identical batch size and otherwise consistent environment for all architectures with *N* = 5 repetitions. *MoNet* significantly outperformed both *U-Net-64* and *Attention U-Net* with regards to inference and training time. Results are shown in Table 2.

**Table 2:** CPU inference time (sec) for a CT scan of 150 slices and timer per batch (sec) on GPU, both at a resolution of 256 × 256.

Architecture	Inference times(s) (CPU)	Time per batch(s) (GPU)
*U-Net-64*	45.34±1.77	0.747±0.30
*U-Net-16*	**7.03±0.21**	**0.152±0.11**
*Attention U-Net*	53.30±0.53	0.905±0.12
*MoNet (ours)*	14.88±0.32	0.462±0.16

### Serialized model size as an indicator for network traffic in federated learning

We performed a comparison of the size taken up by the weights of *MoNet* and the other *U-Net* like architectures. Federated learning requires the frequent transfer of parameter updates over a network, hence the serialized model size of a given architecture can serve as an estimate of the amount of network traffic generated when deployed in a federated learning application. *MoNet* with its small number of parameters is significantly smaller in size than *U-Net-16* and an order of magnitude smaller than *U-Net-64* and *Attention U-Net*. Results are shown in Table 3.

**Table 3:** Comparison of storage space occupied by *MoNet* and other *U-Net* variants.

Architecture	Parameter count	Size in memory
*U-Net-64*	31, 054, 145	118.6 MB
*U-Net-16*	1, 946, 705	7.6 MB
*MoNet (ours)*	**403, 556**	**1.8 MB**

### Visualization of intermediate activations

**Fig 5:**
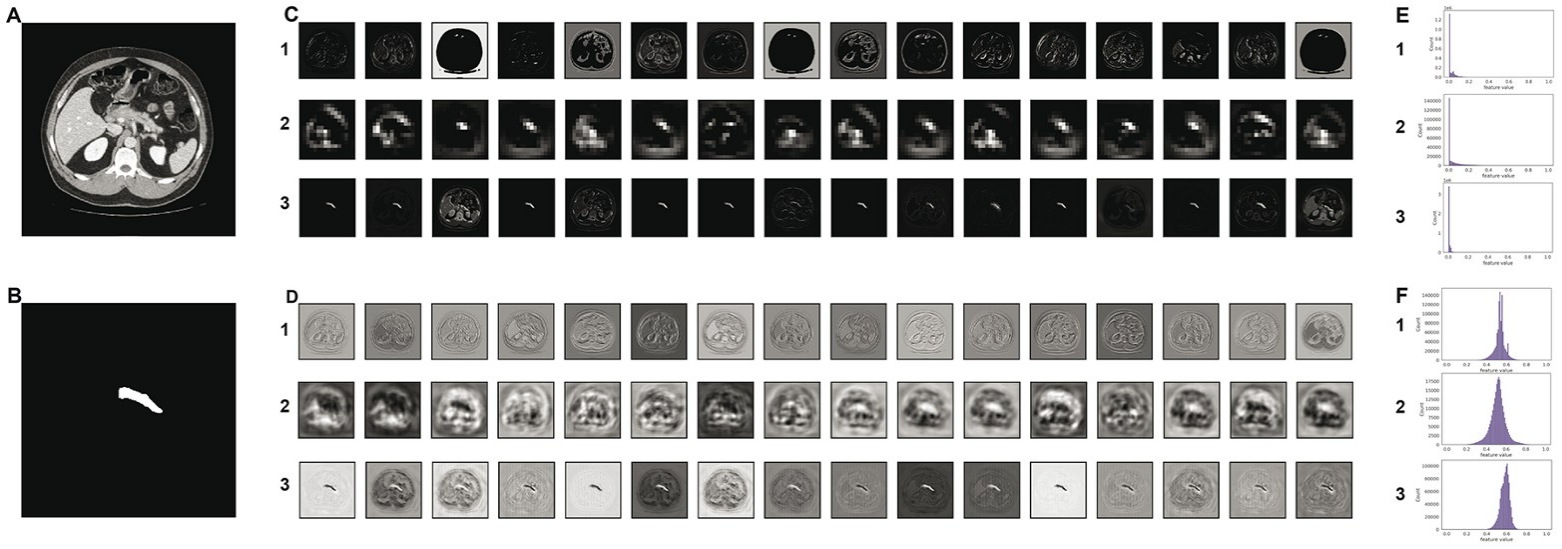
Input image (A) and target (B) alongside visualizations of the first 16 channels of intermediate activations for the given input image produced by early (1), middle (2) and late (3) convolutional layers in *U-net* (C) and *MoNet* (D). Histograms computed for all channels in the feature maps for early (1), middle (2), and late (3) convolution layers for *U-net* (E) and *MoNet* (F).

To corroborate our hypothesis that *MoNet* achieves superior semantic segmentation performance due to an improved utilization of its convolutional filters, leading to more information-rich feature maps, we examined differences between the features extracted by *U-Net-64* and *MoNet* at early, intermediate and late convolutional layers. *MoNet* extracts feature maps with overall higher resolution. Moreover, we found the filter activations at all examined layers of *U-Net-64* to collapse to a region near zero. We thus assume that many of these filters remain essentially unutilised. On the contrary, *MoNet* produced non-zero activations at all examined layers. These results are presented in Fig 5.

## Discussion

We present an efficient, high-performance *U-Net*-like segmentation algorithm and show a substantial reduction in parameter count and expected network traffic in federated learning applications (indicated by serialized model size). Compared to *U-Net-64* and *Attention U-Net*, our method achieves a substantial inference latency reduction on CPU hardware, enabling remote diagnosis applications in centers without GPUs. Furthermore we show reduced training time on GPU, which could benefit federated training as well as swift fine-tuning when model personalisation is required. Both are made possible by our method while exceeding or matching the segmentation performance of all other evaluated algorithms. We thus believe our architecture to be a promising candidate for utilization in large-scale collaborative medical imaging workflows and particularly in resource constrained environments.

We chose the tasks of pancreatic segmentation and brain tumor segmentation due to the poor prognosis and increasing incidence of PDAC [30, 31] and the typically dismal prognosis of brain tumors [32], both of which mandate the development of enhanced diagnosis and treatment strategies. Our recent findings suggest that quantitative image analysis can identify molecular subtypes related to different response to chemotherapeutic drugs [33] or predict patient survival [34] in PDAC. In all quantitative imaging workflows, automated region-of-interest definition increases the reliability and validity of such findings, and offers substantial time savings compared to manual expert-based segmentation. However, the success of automated segmentation algorithms is constrained by the findings’ poor differentiability from adjacent structures of similar attenuation/signal, variability in position of the segmentation target and alterations due to pathology such as edema or other inflammatory changes. Existent work in deep learning-assisted semantic segmentation of medical images and the pancreas in particular has focused on expanding previously available architectures such as the *U-Net* [21] into the three-dimensional context [24] or on improving segmentation results by incorporating attention mechanisms into the architecture [29]. Other approaches have used complex ensembles of 2D and 3D models to extract the maximum amount of information in the CT images [16]. All these modifications however result in a further increase in the (already substantial) computational requirements of these architectures, rendering such *U-Net* derivatives impractical for the utilization in the above-mentioned decentralized learning applications. In other application domains, i.e. image classification, MobileNet and EfficientNet have demonstrated strong performance combined with high computational efficiency. MobileNet achieves this through the utilisation of depth-wise separable convolutions and EfficientNet through optimal trade-offs between model depth and width. In contrast, our method exploits properties of the feature space specific to semantic segmentation through the utilization of higher resolution feature maps in the *bottleneck* section of the network, and thus enables competitive segmentation performance with the state-of-the-art while offering substantial efficiency gains. Recent work on semantic segmentation provides evidence in favor of architectures performing image feature extraction at multiple scales by utilizing dilated convolutions instead of relying merely on the scale-decreasing backbones employed in traditional fully convolutional architectures [19, 35–37]. Our work corroborates this notion, since multi-scale feature extraction combined with larger receptive fields at the same hierarchical level seem to capture both more robust and higher quality features compared to the fixed kernel size design encountered in traditional *U-Net*-like architectures. Moreover, architectures with several down-sampling operations and/or many filters such as the *U-Net* (with 4 down-sampling stages) cannot leverage the large number of parameters sufficiently well to warrant their utilization at least in medical imaging tasks, typically characterized by small segmentation targets (such as the pancreas or small tumors). This is substantiated by our results from *U-Net-64*’s activation histograms, which were concentrated at a near-zero region.

Our results indicate that *MoNet* extracts more robust features that generalize better to out-of-sample data than the compared methods, as shown by *MoNet*’s performance on the independent validation set and the activation histograms. The poor performance of the 64 filter *U-Net* and *Attention U-Net* in the out-of-sample generalization challenge could potentially be caused by the overparameterization of these architectures, making them prone to over-fitting the data-generating distribution of the training data, while the two smaller models(*U-Net-16* and *MoNet*) tested seemed to generalize better to the out-of-sample data, supporting this hypothesis.

Our work is not without limitations. The generalizability of our findings should be confirmed using larger, multi-institutional training and validation sets. Furthermore, we only compared our algorithm against models based on the use of a single 2D *U-Net*-style network. Algorithms such as *nnU-Net* [16] based on *U-Net* ensembles offer superior performance, however at the expense of extremely high computational and post-processing requirements and thus much slower inference times (especially on CPU). Furthermore implementing and establishing a real-world federated learning application was out of scope for this study and will be addressed in future work.

## Conclusion

In conclusion, we propose an optimized semantic segmentation algorithm with small size and low inference latency, particularly suited for decentralized applications such as federated learning. Our work can benefit both, radiological research and clinical translation of artificial intelligence workflows in medical imaging by providing consistent, high-quality segmentation for machine learning tasks.
